# The complete mitochondrial genome of Jinbian carp *Cyprinus carpio* (Cypriniformes: Cyprinidae)

**DOI:** 10.1080/23802359.2018.1495126

**Published:** 2018-10-25

**Authors:** Xiangchen Ye, Yejian Lv, Lingjing Wei, Jie Huang, Yanhong Wen, Guijiao Zhang, Sheng Zhang, Zhushan Yang, Kang Liu

**Affiliations:** a Aquatic Species Introduction and Breeding Center of Guangxi, Nanning, China;; b Extension Station of Fisheries Technology of Liuzhou, Liuzhou, China;; c Fisheries Technology Extension Station of Rongshui Miaozu Autonomous County, Liuzhou, China

**Keywords:** Mitochondrial genome, Jinbian carp, *Cyprinus carpio*, Cyprinidae

## Abstract

Jinbian carp (*Cyprinus carpio*) is an endemic species in China. The complete mitochondrial genome of Jinbian carp is determined to be 16,581 bp in length and includes 13 protein-coding genes, 2 ribosomal RNA genes, 22 transfer RNA genes, and a control region. Its structural organization and gene order are equivalent to other common carp strains. The phylogenetic analyses will contribute to further insights of the taxonomy and phylogeny in Cyprinidae family.

Common carp is old freshwater fish that have been cultured as aquaculture species all over the world for thousand years (FAO 2004). Jinbian carp (*Cyprinus carpio*) is an endemic species that mainly distributed in Guangxi Province, China. It has two golden stripes on both sides of its Dorsal fin, which can be used to distinguish from other carp varieties. Due to its high-protein, low-fat, easy culture, and fast growth, Jinbian carp was regularly cultured in rice paddy and ponds as an ecological friendly agriculture-aquaculture form in Southwest China. To better define the phylogenetic relationship between Jinbian carp and other closely related varieties, we cloned and analyzed its complete mitochondrial genome sequence for the first time.

In this study, the Jinbian carp was sampled from Rongshui (25°04′N, 109°14′E), Guangxi province, China. Fin tissues were preserved in 95% ethyl alcohol. The extracted DNA is stored in the laboratory of Aquatic Species Introduction and Breeding Center of Guangxi, China. Seventeen pairs of primers and DNA samples were used to amplify the complete mitogenome using PCR-based method (Mabuchi et al. 2006; Zhang et al. 2009).

The mitogenome sequence of Jinbian carp was 16,581 bp in length (GenBank accession *No.* MH202953) and consisted of 13 protein-coding genes (PCGs), 22 tRNAs, 2 rRNAs, and a control region (D-loop). The overall base composition was 31.92% A, 15.73% G, 24.82% T, and 27.53% C, with a slight A + T bias. In the 13 PCGs, ND6 gene especially stayed on the L-strand while other genes were on the H-strand. Most of PCGs started with ATG while CO1 genes used GTG as start codon. ND2 and ND3 genes were used TAA as the stop codon, ND4 and cytb genes were ended with incomplete stop codon of T. The structural organization and gene order of mtDNA sequence of Jinbian carp was similar to other common carp strains (Chang et al. 1994; Wang et al. 2013; Liu et al. 2016).

A phylogenetic tree was constructed to validate the phylogenetic position of Jinbian carp. MEGA package was used to construct a Neighbor-joining tree containing complete sequences of Jinbian carp and other 19 Cyprinidae varieties downloaded from GenBank (Figure 1). *Cabdio morar* was used as an outgroup member. The tree showed that Jinbian carp was a group with other *Cyprinus carpio* in Cyprinidae family and closely related to Zujiang wild carp.

**Figure 1.**
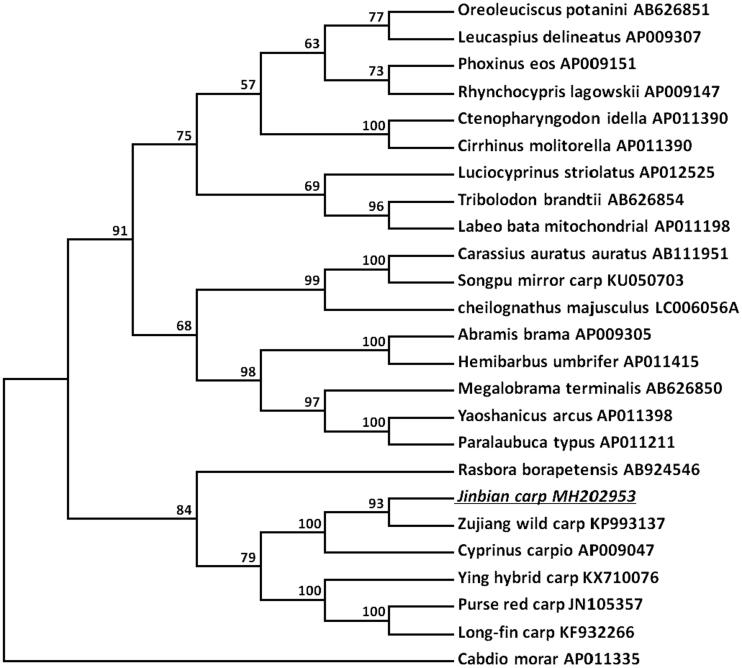
Molecular phylogeny of Jinbian carp (*Cyprinus carpio*) and other Cyprinidae varieties based on complete mitogenome. The mtDNA sequences are downloaded from Genbank and the phylogenic tree is constructed by Neighbor-joining method with 1000 bootstrap replicates.
